# Adiponectin-leptin Ratio is a Functional Biomarker of Adipose Tissue Inflammation

**DOI:** 10.3390/nu11020454

**Published:** 2019-02-22

**Authors:** Gema Frühbeck, Victoria Catalán, Amaia Rodríguez, Beatriz Ramírez, Sara Becerril, Javier Salvador, Inmaculada Colina, Javier Gómez-Ambrosi

**Affiliations:** 1 Metabolic Research Laboratory, Clínica Universidad de Navarra, 31008 Pamplona, Spain; gfruhbeck@unav.es (G.F.); vcatalan@unav.es (V.C.); arodmur@unav.es (A.R.); bearamirez@unav.es (B.R.); sbecman@unav.es (S.B.); 2 CIBER Fisiopatología de la Obesidad y Nutrición (CIBEROBN), Instituto de Salud Carlos III, 31008 Pamplona, Spain; jsalvador@unav.es; 3 Obesity and Adipobiology Group, Instituto de Investigación Sanitaria de Navarra (IdiSNA), 31008 Pamplona, Spain; icolina@unav.es; 4 Department of Endocrinology & Nutrition, Clínica Universidad de Navarra, 31008 Pamplona, Spain; 5 Department of Internal Medicine, Clínica Universidad de Navarra, 31008 Pamplona, Spain

**Keywords:** adiponectin/leptin ratio, adipose tissue, obesity, type 2 diabetes, metabolic syndrome, inflammation

## Abstract

Obesity favors the development of cardiometabolic alterations such as type 2 diabetes (T2D) and the metabolic syndrome (MS). Obesity and the MS are distinguished by an increase in circulating leptin concentrations, in parallel to a drop in the levels of adiponectin. Consequently, the Adpn/Lep ratio has been suggested as a maker of dysfunctional adipose tissue. We aimed to investigate in humans (*n* = 292) the reliability of the Adpn/Lep ratio as a biomarker of adipose tissue dysfunction. We considered that an Adpn/Lep ratio of ≥1.0 can be considered normal, a ratio of ≥0.5 <1.0 suggests moderate-medium increased risk, and a ratio of <0.5 indicates a severe increase in cardiometabolic risk. Using these cut-offs, 5%, 54% and 48% of the lean, normoglycemic and without-MS subjects, respectively, fall within the group with an Adpn/Lep ratio below 0.5; while 89%, 86% and 90% of the obese, with T2D and with MS patients fall within the same group (*p* < 0.001). A significant negative correlation (*r* = −0.21, *p* = 0.005) between the Adpn/Lep ratio and serum amyloid A (SAA) concentrations, a marker of adipose tissue dysfunction, was found. We concluded that the Adpn/Lep ratio is a good indicator of a dysfunctional adipose tissue that may be a useful estimator of obesity- and MS-associated cardiometabolic risk, allowing the identification of a higher number of subjects at risk.

## 1. Introduction

Obesity has become, over recent decades, the most prevalent metabolic alteration, constituting one of the main causes of death and disability [1]. According to a recent study, more than 700 million people in the world were obese by 2015, and the prevalence of obesity has doubled in more than 70 countries since 1980 [2]. Obesity increases the risk of developing cardiometabolic alterations such as type 2 diabetes (T2D), hypertension, dyslipidemia, and non-alcoholic fatty liver disease (NAFLD), endangering the health improvements achieved in recent decades and producing an increase in morbidity [3,4].

Obesity is defined medically as a condition of excessive accumulation of adipose tissue, of sufficient extent to produce adverse health consequences [5]. However, the molecular mechanisms involved in the development of obesity and the expansion of adipose tissue have not been fully clarified [6,7]. Only a couple of decades ago adipose tissue was considered a passive organ for excess energy storage as triglycerides [8]. However, in the last years adipose tissue has been shown to behave as a highly active endocrine organ, based on its ability to secrete a wide variety of biologically active adipokines, such as leptin, adiponectin, tumor necrosis factor-α (TNF-α), or interleukin-6 (IL-6), which are known to be involved in different physiological processes [7]. The development of dysfunctional adipose tissue, characterized by unresolved inflammation, together with altered extracellular matrix remodeling and impaired angiogenesis has been proposed as a key event to explain the development of obesity-associated cardiometabolic alterations [9]. It is now accepted that obesity and the accompanying chronic unresolved inflammation are associated with an imbalance in homeostatic mechanisms regulating metabolism, leading to adipose tissue dysfunction, characterized by altered secretion of adipokines and negatively affecting the correct function of adipose tissue leading to ectopic fat accumulation and lipotoxicity [10].

It is well recognized that adipokines play an important role in the pathophysiological link between dysfunctional adipose tissue and cardiometabolic alterations [7,11,12]. Leptin is primarily produced by adipose tissue in proportion to the amount of body fat stores being involved in the regulation of food intake, energy homeostasis and other physiological processes [13,14]. Adiponectin is also secreted almost exclusively by adipocytes, and decreases in obese patients. This adipokine protects against insulin resistance and excessive hepatic lipid accumulation with anti-inflammatory effects [15,16]. Both leptin and adiponectin have been related to cardiometabolic risk factors [12,17,18,19,20]. The adiponectin/leptin (Adpn/Lep) ratio has been proposed as a marker of adipose tissue dysfunction [21,22]. This emerging biomarker correlates with insulin resistance better than adiponectin or leptin alone being significantly reduced in patients with the metabolic syndrome (MS) [19]. Furthermore, the Adpn/Lep ratio decreases with increasing number of metabolic risk factors for MS [21], having been proposed as a predictive marker for the MS [21,22]. Moreover, the Adpn/Lep ratio is negatively correlated with markers of low-grade chronic inflammation, such as C-reactive protein (CRP) [22]. High circulating leptin levels, as those found in obese individuals, together with an impaired response to the anorectic effects of leptin is suggestive of leptin resistance. Subjects with low adiponectin concentrations may lose its cardioprotective and anti-inflammatory actions. Therefore, the Adpn/Lep ratio represents a marker suggestive of the pathophysiological function of both adipokines [22]. In this sense, an increase in this ratio has been related with reduced atherosclerosis risk as well as with a decreased risk of some types of cancer in epidemiological studies [22]. We previously proposed several cut-off points that may be useful in the detection of a dysfunctional adipose tissue with potential metabolic repercussion [22].

In the present study, we aimed to investigate in humans the reliability of the Adpn/Lep ratio as a biomarker of adipose tissue dysfunction. We studied the anthropometric and metabolic characteristics of patients classified according to the proposed cut-off points of the Adpn/Lep ratio, as well as its relation with obesity, glycemic status and the MS. In addition, we analyzed whether the Adpn/Lep ratio reflects adipose tissue dysfunction by studying its association with serum amyloid A (SAA), which is a validated marker of dysfunctional adipose tissue [23], and by comparison with another indicator of adipose tissue dysfunction which is the visceral adiposity index (VAI) [24].

## 2. Subjects and Methods

### 2.1. Study Participants

We conducted a cross-sectional analysis of 292 patients (135 men and 157 women) aged 18–82 years, with similar socio-economical characteristics, including patients visiting the Department of Endocrinology and Nutrition of the Clínica Universidad de Navarra (Pamplona, Spain) for weight loss treatment, as well as hospital and University staff undergoing an annual routine health check-up. Normal weight was considered as having a body mass index (BMI) <25 kg/m^2^, while obesity was defined as having a BMI ≥30 kg/m^2^ following the World Health Organization criteria [3]. Subjects were additionally classified as having normoglycemia (NG), impaired fasting glucose (IFG) or type 2 diabetes (T2D) following the criteria of the American Diabetes Association [25] based on fasting plasma glucose. Glucose intolerance or T2D was of recent-onset being diagnosed during the study and, therefore, participants were not on antidiabetic medication or insulin therapy. For three individuals, glucose levels were not available. The presence or absence of the MS was based on the harmonized criteria [26]. Forty-six subjects could not be classified according to MS in most cases because blood pressure or waist circumference were missed. Individuals with signs of infection were excluded. According to previous data, we considered that an Adpn/Lep ratio equal or higher to 1.0 (with adiponectin concentrations expressed in μg/mL and leptin levels in ng/mL) can be considered normal, a ratio between 0.5 and 1.0 can indicate moderate-medium increased risk, and a ratio below 0.5 suggests a severe increase in cardiometabolic risk [22]. The experimental design was approved by the Research Ethics Committee of the University of Navarra and the study was performed in accordance with the ethical standards as laid down in the Declaration of Helsinki and its later amendments. Volunteers gave their informed consent to participate in the study.

### 2.2. Anthropometry

The anthropometric determinations, as well as the blood extractions, were performed on a single day. Height was measured to the nearest 0.1 cm with a Holtain stadiometer (Holtain Ltd., Crymych, UK), while body weight was measured with a calibrated electronic scale to the nearest 0.1 kg with subjects wearing a swimming suit and cap. BMI was calculated as weight in kg divided by the square of height in meters. Percentage of body fat was estimated using the Clínica Universidad de Navarra-Body Adiposity Estimator (CUN-BAE) [27]. Waist circumference was measured at the midpoint between the iliac crest and the rib cage on the midaxillary line. Blood pressure was measured after a 5-minute rest in the semi-sitting position with a sphygmomanometer (A&D Instruments Ltd., Abingdon, UK). Blood pressure was determined at least 3 times at the right upper arm and the mean was used in the analyses.

### 2.3. Serum Biochemistry

Blood samples were collected after an overnight fast in the morning in order to avoid potential confounding influences due to hormonal rhythmicity. Plasma glucose was analyzed by an automated analyzer (Roche/Hitachi Modular P800, Basel, Switzerland) as previously described [28]. Insulin was measured by means of enzyme-amplified chemiluminescence assay (Immulite 2000, Siemens AG, Erlangen, Germany). Indirect measures of insulin resistance and insulin sensitivity were calculated by using the homeostatic model assessment (HOMA) and the quantitative insulin sensitivity check index (QUICKI), respectively. Total cholesterol and triglyceride concentrations were determined by enzymatic spectrophotometric methods (Roche, Basel, Switzerland). Serum high-density lipoprotein cholesterol (HDL-C) was quantified by a colorimetric method in a Beckman Synchron**^®^** CX analyzer (Beckman Instruments, Ltd., Bucks, UK). Low-density lipoprotein (LDL-C) was calculated by the Friedewald formula. High-sensitivity C-reactive protein (CRP) was measured using the Tina-quant CRP (Latex) ultrasensitive assay (Roche). Fibrinogen concentrations were determined according to the method of Clauss using a commercially available kit (Hemoliance**^®^**, Instrumentation Laboratory, Barcelona, Spain). Homocysteine was determined applying a fluorescence polarization immunoassay (Axis Biochemicals ASA, Oslo, Norway) using an IMX analyzer (Abbott, Abbott Park, IL, USA). White blood cell (WBC) count was measured using an automated cell counter (Beckman Coulter, Fullerton, CA, USA). Uric acid, alanine aminotransferase (ALT), aspartate aminotransferase (AST), γ-glutamyltransferase (γ-GT), and creatinine were measured by enzymatic tests (Roche) in an automated analyzer (Roche/Hitachi Modular P800). The AST/ALT ratio was calculated as an indirect indicator of hepatic steatosis and fatty liver disease [29]. Leptin levels were quantified by a double-antibody radioimmunoassay method (Linco Research, Inc., St. Charles., MO, USA) as previously described [30]; intra-and inter-assay coefficients of variation were 5.0% and 4.5%, respectively. Adiponectin was measured by enzyme-linked immunosorbent assay (ELISA) (BioVendor, Brno, Czech Republic); intra-and inter-assay coefficients of variation were 6.7% and 7.8%, respectively. SAA was determined by ELISA (BioSource International Inc., Camarillo, CA, USA). Intra-and inter-assay coefficients of variation were 4.9% and 7.9%, respectively. The marker of dysfunctional adipose tissue VAI was calculated as previously established [31].

### 2.4. Statistical Analysis

Data are presented as mean ± standard deviation (SD) unless otherwise indicated. Differences between groups were analyzed by one-way ANOVA followed by Fisher’s least significant difference (LSD) tests or two-tailed unpaired Student’s *t* tests, as appropriate. Differences in distribution regarding the Adpn/Lep ratio cut-offs and each metabolic condition (obesity, T2D or MS) were analyzed by χ^2^ analysis. Correlations between two variables were computed by Pearson’s correlation coefficients (*r*). The calculations were performed using SPSS 23 (SPSS, Chicago, IL, USA) and GraphPad Prism 6 (GraphPad Software, Inc., La Jolla, CA, USA). A *p* value lower than 0.05 was considered statistically significant.

## 3. Results

Anthropometric and biochemical characteristics of the individuals included in the study according to the Adpn/Lep ratio are shown in Table 1. From the whole cohort, 135 (46%) were males and 157 (54%) were females, with no differences in gender distribution (*p* = 0.374). A high proportion of the subjects included in the study (*n* = 209, 72%) were classified as obese, while 83 individuals (28%) were considered lean according to BMI. According to the Adpn/Lep ratio, 51 subjects had a ratio equal or higher to 1.0, 50 individuals exhibited a ratio equal or higher to 0.5 and lower than 1.0, and 191 subjects presented a ratio lower than 0.5. Body adiposity, BMI and waist circumference were significantly higher (*p* < 0.001) in the ≥0.5–<1.0 and <0.5 groups as compared to the ≥1.0 group. Glucose and lipid profiles were significantly worse only in the <0.5 group with the exception of QUICKI that was also decreased in the ≥0.5–<1.0 group as compared to the ≥1.0 group. Uric acid and markers of inflammation such as CRP and fibrinogen levels were significantly increased (*p* < 0.001) in the <0.5 group, while WBC count was significantly increased in both the ≥0.5–<1.0 and <0.5 groups as compared to the ≥1.0 group. The ratio AST/ALT, a marker of hepatic steatosis, was significantly reduced in the ≥0.5–<1.0 group, being further decreased in the <0.5 group (*p* < 0.001). Although there were differences regarding age, they were due to the older age of subjects in the ≥0.5–<1.0 group, while those in the ≥1.0 and the <0.5 group were of similar age.

**Figure 1:**
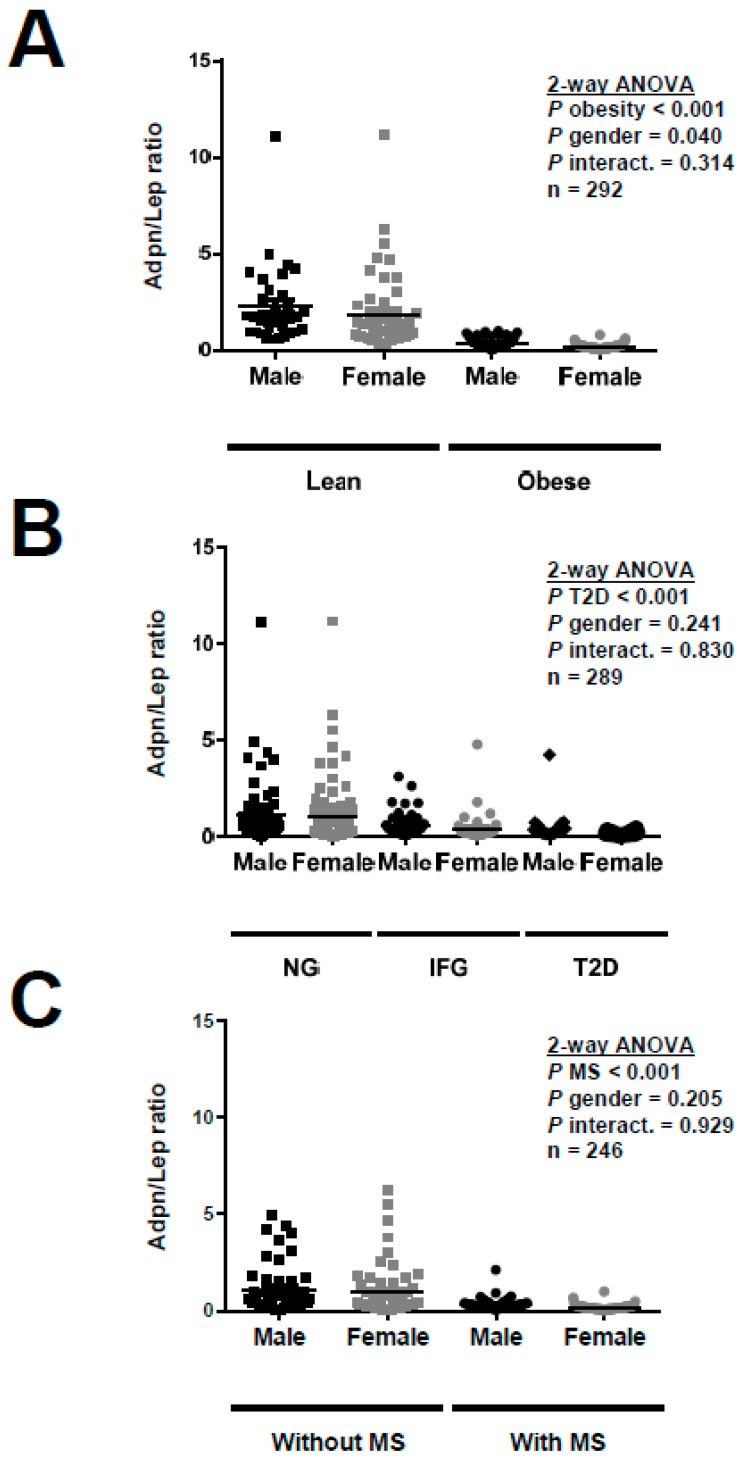
Adiponectin/Leptin (Adpn/Lep) ratio according to gender and (**A**) obesity (*n* = 292), (**B**) type 2 diabetes (*n* = 289) and (**C**) metabolic syndrome (*n* = 246). Differences between groups were analyzed by two-way ANOVA. NG, normoglycemia; IFG, impaired fasting glucose; T2D, type 2 diabetes; MS, metabolic syndrome.

The Adpn/Lep ratio was significantly lower in individuals with obesity (Lean: 2.00 ± 1.98; Obese: 0.26 ± 0.19; *p* < 0.001) as can be observed in Figure 1A. Taking into account only the lean subjects, the Adpn/Lep ratio was significantly decreased in individuals with high adiposity (low adiposity, *n* = 42: 2.54 ± 2.43; high adiposity, *n* = 41: 1.47 ± 1.18; *p* = 0.012, Figure S1). Moreover, in an analysis of proportions (Table 2) 35% and 5% of lean subjects had an Adpn/Lep ratio in the ≥0.5–<1.0 and <0.5 groups, respectively, while regarding obese individuals the percentages were 10% and 89%, respectively, for the same groups (*p* < 0.001). Regarding T2D, the Adpn/Lep ratio was significantly lower in individuals with impaired fasting glucose (0.49 ± 0.69) and further decreased in patients with T2D (0.37 ± 0.68) as compared to the normoglycemic ones (1.03 ± 1.65) (*p* < 0.001 for the general comparison; with post hoc significant differences for IFG and T2D groups vs. NG *p* < 0.01 for both) as shown in Figure 1B. From the subjects with normoglycemia 21% and 54% had an Adpn/Lep ratio in the ≥0.5–<1.0 and <0.5 groups, respectively, while regarding individuals with T2D the percentages were 11% and 86%, respectively, for the same groups (*p* < 0.001). The Adpn/Lep ratio was significantly lower in individuals with MS (Without MS: 1.01 ± 1.24; With MS: 0.26 ± 0.24; *p* < 0.001) as can be seen in Figure 1C. From the subjects without the MS 26% and 48% had an Adpn/Lep ratio in the ≥0.5–<1.0 and <0.5 groups, respectively, while in individuals with the MS the percentages were 9% and 90%, respectively, for the same groups (*p* < 0.001). No significant differences in the Adpn/Lep ratio regarding gender (males 0.81 ± 1.30, females 0.72 ± 1.35; *p* = 0.542) were found.

**Figure 2:**
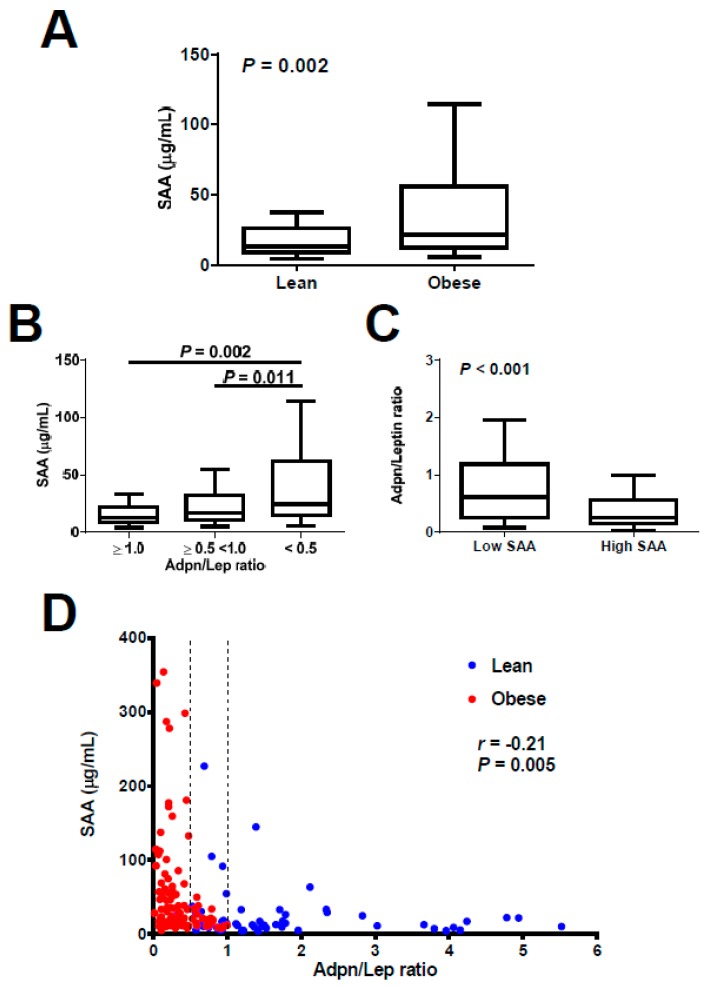
(**A**) Comparison of serum serum amyloid A (SAA) concentrations between lean (*n* = 60) and obese (*n* = 111) individuals. Statistical differences were analyzed by two-tailed unpaired Student’s *t* test. (**B**) Serum levels of SAA according to Adiponectin/Leptin (Adpn/Lep) ratio: ≥1.0 (*n* = 36), ≥0.5 <1.0 (*n* = 41) and <0.5. (*n* = 94). Statistical differences were analyzed by one-way ANOVA followed by Fisher’s LSD test. Box in (**A**) and (**B**) represents interquartile range and median inside, with whiskers plotted according to the Tukey method. (**C**) Adpn/Lep ratio according to serum concentrations of SAA (low SAA, *n* = 104; high SAA *n* = 67). Statistical differences were analyzed by two-tailed unpaired Student’s *t* test. (**D**) Scatter diagram showing the correlation between circulating concentrations of the Adpn/Lep ratio and SAA. Pearson’s correlation coefficient and *p* value are indicated. Vertical discontinuous lines denote the Adpn/Lep ratio proposed cut-offs of 0.5 and 1.0.

We next aimed to analyze the reliability of the Adpn/Lep ratio as a marker of adipose tissue dysfunction. To do so we used serum levels of SAA as an indirect signal of dysfunctional adipose tissue [23]. From the 171 subjects with serum concentrations of SAA available, SAA levels were significantly increased in the individuals with obesity as compared to lean subjects (Lean, *n* = 60, 25.2 ± 36.3, Obese, *n* = 111, 50.9 ± 69.1 μg/mL; *p* = 0.002; Figure 2A). Moreover, SAA concentrations were significantly increased in the <0.5 group (*n* = 94, 56.5 ± 73.7 μg/mL) as compared to the ≥0.5–<1.0 (*n* = 41, 28.3 ± 38.1 μg/mL; *p* = 0.011) and ≥1.0 (*n* = 36, 19.4 ± 24.5 μg/mL; *p* = 0.002) groups, as shown in Figure 2B. Conversely, after splitting the sample in those with low SAA levels (below 25.0 μg/mL, cutoff which encompass 75% of normal weight subjects) or high concentrations of SAA (equal or higher than 25.0 μg/mL) we found that those with high SAA levels showed a significantly decreased Adpn/Lep ratio (low SAA, *n* = 104, 1.01 ± 1.19, high SAA, *n* = 67, 0.48 ± 0.53; *p* < 0.001; Figure 2C). A significant negative correlation between the Adpn/Lep ratio and SAA levels (*r* = −0.21, *p* = 0.005) was found (Figure 2D). Taking into account only those patients with obesity, a significant negative correlation between the Adpn/Lep ratio and SAA concentrations (*r* = −0.23, *p* = 0.017) was still found. Finally, after adjustment for BMI, the correlations of the Adpn/Lep ratio with anthropometric markers and markers of inflammation and fatty liver remained, although attenuated, but those with markers of glucose metabolism vanished with the exception of QUICKI (Table S1).

Finally, to further strengthen the reliability of the Adpn/Lep ratio as a marker of adipose tissue dysfunction we compared the degree of correlation with several anthropometric and metabolic variables of the Adpn/Lep ratio and another marker of dysfunctional adipose tissue, the VAI [24,31]. Interestingly, the correlation of the Adpn/Lep ratio with BMI (*r* = −0.49, *p* < 0.001), body adiposity (*r* = −0.56, *p* < 0.001) and waist circumference (*r* = −0.51, *p* < 0.001) was higher than that of the VAI with BMI (*r* = 0.30, *p* < 0.001), body adiposity (*r* = 0.27, *p* < 0.001) and waist circumference (*r* = 0.35, *p* < 0.001), although VAI is calculated with waist circumference, BMI, triglycerides (TG) and HDL (Table 3). VAI presented better correlations with markers of glucose and lipid metabolism, with the exception of LDL, which was only correlated with the Adpn/Lep ratio (*r* = −0.14, *p* = 0.031) despite VAI being calculated with a pair of lipid variables, as mentioned above. Importantly, the Adpn/Lep ratio was better correlated with the markers of inflammation CRP, WBC and fibrinogen than VAI as well as with the AST/ALT ratio, a marker of hepatic steatosis. The Adpn/Lep ratio was correlated with serum SAA concentrations (*r* = −0.18, *p* = 0.034), while VAI showed no significant correlation with SAA levels (*r* = 0.10, *p* = 0.245). Finally, the Adpn/Lep ratio showed a significant negative correlation with VAI (*r* = −0.26, *p* < 0.001).

## 4. Discussion

The main finding of the present study is that the Adpn/Lep ratio is a reliable biomarker of adipose tissue dysfunction, as evidenced by its association with serum concentrations of SAA and the comparison with VAI, another marker of dysfunctional adipose tissue. Accordingly, the proportion of subjects with an Adpn/Lep ratio below the lower cut-off point proposed (and considered as of high cardiometabolic risk) is significantly higher in individuals with obesity, T2D or the MS.

We have recently shown that a low Adpn/Lep ratio is suggestive of a dysfunctional adipose tissue and that patients with reduced ratio are characterized by an increased cardiometabolic risk evidenced by raised systemic inflammation and oxidative stress [19]. We found a strong negative correlation of the Adpn/Lep ratio with circulating concentrations of CRP, which suggested that this marker may be reflecting adipose tissue dysfunction-induced systemic inflammation in the context of the MS [19]. Consequently, we proposed that dysfunctional adipose tissue, indicated by a low Adpn/Lep ratio, may represent a scenario of raised proinflammatory factors as potential mediators in the ethiopathogenesis of the MS [19].

In the present study we provided evidence that the Adpn/Lep ratio is a reliable marker of adipose tissue dysfunction since it is highly correlated with the serum concentrations of SAA. Increased circulating SAA levels in obesity may be the result of increased expression and secretion from dysfunctional adipose tissue [23]. The expanded mass of dysfunctional adipose tissue in obesity is reportedly a source of several proinflammatory molecules, such as TNF-α, IL-6 and MCP-1, which are predominantly produced by the stromal-vascular cells (SVCs) within adipose tissue [10]. By contrast, SAA, like adiponectin and leptin, is expressed and secreted by adipocytes and not SVCs stimulating the release of proinflammatory cytokines in adipose SVCs [32]. For this reason, it has been considered that SAA acts as an autocrine/paracrine factor to upregulate the secretion of proinflammatory factors by SVCs and has been considered to be a local and systemic proinflammatory adipokine, rather than just a marker of inflammation [23,33]. However, SAA is not routinely used in clinical metabolic studies, since the levels of leptin and adiponectin are available more frequently than SAA and, given that SAA may behave as an acute phase protein, in some cases its levels may not reflect adipose tissue inflammation [33]. Serum concentrations of SAA are significantly increased in obese subjects as well as in those individuals with an Adpn/Lep ratio lower than 0.5, regardless of their obese state. In addition, we found a negative correlation of the Adpn/Lep ratio with SAA levels, which confirms that the Adpn/Lep ratio may be considered a good estimator of adipose tissue inflammation and, therefore, adipose tissue dysfunction. Moreover, while another proposed indicator of adipose tissue dysfunction, namely VAI, was better correlated with glucose and lipid metabolism markers (taking into consideration that is calculated from anthropometric and lipid variables), the Adpn/Lep ratio was better correlated with BMI, waist circumference and blood pressure, as well as with markers of systemic inflammation and hepatic steatosis. Thus, we considered that the Adpn/Lep ratio is a better marker of dysfunctional adipose tissue than VAI. Moreover, our data suggest that the Adpn/Lep ratio may be suggestive of the presence of NAFLD. This should be confirmed in further studies.

Since the Adpn/Lep ratio reflects the dysfunction of adipose tissue, we and others have postulated that this ratio may be clinically useful to identify subjects susceptible to cardiometabolic diseases [19,21,22,34]. We proposed cut-off points that may be predictive of cardiometabolic risk according to the Adpn/Lep ratio (with adiponectin concentrations expressed in μg/mL and leptin levels in ng/mL in fasting conditions) [22]. Classifying the subjects included in the present study with those cut-offs shows that 5%, 54% and 48% of the lean, normoglycemic and without-MS subjects fall within the group with an Adpn/Lep ratio below 0.5; while 89%, 86% and 90% of the obese, with T2D and with MS patients fall within the same group. This indicates that the Adpn/Lep ratio allows the identification of a higher number of subjects at cardiometabolic risk. Our data show that the Adpn/Lep ratio is more strongly correlated with anthropometric markers such as BMI, body adiposity and waist circumference than with markers of metabolism or inflammation, something that is not unexpected, since the Adpn/Lep ratio is calculated after leptin levels which correlate highly with body adiposity. However, although the Adpn/Lep ratio is more related to obesity than to the metabolic state, we have shown that it is a good proxy of adipose tissue inflammation. In this sense, a low Adpn/Lep ratio indicates a dysfunctional adipose tissue that may be accompanied by alterations in metabolism in the case of well-established obesity or may be suggesting a dysfunctional adipose tissue in the first stages of obesity or pre-obesity that, with time, will likely lead to the development of metabolic abnormalities. Larger epidemiological studies will be needed to validate the clinical usefulness of these proposed cutoffs.

Adipose tissue dysfunction with proinflammatory M1 macrophage polarization, altered adipokine profile, insulin resistance and hypoxia, has been related with tumor development [35,36]. Therefore, a low Adpn/Lep ratio could also be used as an estimator of adiposopathy-related cancer risk [34]. In this sense, a high Adpn/Lep ratio has been associated with reduced risk of several cancers [22]. Several therapeutic strategies aimed at increasing the Adpn/Lep ratio have been proposed, mostly focused on the prevention of some types of cancer [22,34]. Besides weight loss, increased physical activity and pharmacology, the nutritional approach is one of the most interesting. Diets rich in fish and omega-3 polyunsaturated fatty acids, as well as fiber supplementation, have been proposed to increase the Adpn/Lep ratio [22].

Some potential limitations of our study should be pointed out. First, our study was conducted with Caucasian subjects and it would need to be determined whether our findings regarding the Adpn/Lep ratio extend to other races. Second, we did not measure adipose tissue inflammation directly and used instead SAA levels as a marker of adipose tissue dysfunction. Third, our study population included 72% obese subjects, supporting the strong association between obesity and adipose tissue inflammation. Further work may consider other populations of subjects with T2D of MS, with a lower proportion of obese subjects, to better discriminate the association of the Adpn/Lep ratio with the presence of metabolic disorders. Future studies directly measuring adipose tissue inflammation or other features of dysfunctional adipose tissue may provide more arguments in favor of the reliability of the Adpn/Lep ratio as a biomarker of adipose tissue dysfunction.

## 5. Conclusions

We concluded that the Adpn/Lep ratio may be considered a good indicator of a dysfunctional adipose tissue, as evidenced by its association with serum concentrations of SAA. The Adpn/Lep ratio, and the proposed cut-off points, may be an interesting and useful estimator of obesity- and MS-associated cardiometabolic risk, allowing the identification of a higher number of subjects at risk.

## Figures and Tables

**Table 1:** Demographic and biochemical characteristics of the individuals classified according to the Adpn/Lep ratio.

Adpn/Lep Ratio	≥1.0	≥ 0.5 <1.0	<0.5	*p*
*n*	51	50	191	
Sex, M/F	25/26	27/23	83/108	0.374
Age, year	48 ± 17	54 ± 16 *	48 ± 14 **†**	0.038
Weight, kg	64 ± 9	77 ± 16 *	91 ± 13 *^,^**†**	<0.001
BMI, kg/m^2^	21.7 ± 2.5	26.9 ± 5.3 *	39.8 ± 7.9 *^,^**†**	<0.001
Body adiposity, %	25.6 ± 6.7	32.8 ± 6.9 *	46.6 ± 7.6 *^,^**†**	<0.001
Waist circumference, cm	79 ± 10	94 ± 14 *	117 ± 15 *^,^**†**	<0.001
SBP, mm Hg	123 ± 26	133 ± 21 *	134 ± 20 *	0.008
DBP, mm Hg	71 ± 10	79 ± 12 *	82 ± 11 *^,^**†**	<0.001
Glucose, mg/dL	91 ± 16	99 ± 27	107 ± 27 *^,^**†**	<0.001
Insulin, μU/mL	4.3 ± 3.1	5.2 ± 3.2	17.3 ± 17.3 *^,^**†**	<0.001
HOMA	1.0 ± 0.7	1.3 ± 0.9	4.7 ± 4.9 *^,^**†**	<0.001
QUICKI	0.41 ± 0.05	0.39 ± 0.05 *	0.33 ±0.04 *^,^**†**	<0.001
Triglycerides, mg/dL	77 ± 41	95 ± 62	140 ± 106 *^,^**†**	<0.001
Total cholesterol, mg/dL	189 ± 39	202 ± 42	204 ± 44	0.084
LDL-cholesterol, mg/dL	111 ± 33	122 ± 35	128 ± 36 *	0.013
HDL-cholesterol, mg/dL	65 ± 16	61 ± 17	48 ± 12 *^,^**†**	<0.001
Uric acid, mg/dL	4.6 ± 1.1	5.1 ± 1.4	6.1 ± 1.4 *^,^**†**	<0.001
CRP, mg/L	1.4 ± 1.9	2.2 ± 2.8	7.6 ± 7.8 *^,^**†**	<0.001
Fibrinogen, mg/dL	191 ± 64	229 ± 93	349 ± 88 *^,^**†**	<0.001
Homocysteine, μmol/L	6.9 ± 2.3	7.8 ± 2.9	10.0 ± 5.4 *	0.028
WBC, 10^6^ cells/mL	5.4 ± 1.3	6.7 ± 1.9 *	6.8 ± 1.6 *	0.003
ALT, U/L	12 ± 7	16 ± 13	26 ± 17 *^,^**†**	<0.001
AST, U/L	14 ± 6	14 ± 7	17 ± 9 *^,^**†**	0.016
AST/ALT ratio	1.34 ± 0.65	1.04 ± 0.41 *	0.75 ± 0.38 *^,^**†**	<0.001
γ-GT, U/L	13 ± 6	20 ± 17	30 ± 28 *^,^**†**	<0.001
Creatinine, mg/dL	0.87 ± 0.15	0.89 ± 0.15	0.85 ± 0.22	0.326
Leptin, ng/mL	5.2 ± 2.9	10.4 ± 6.9	40.9 ± 26.6 *^,^**†**	<0.001
Adiponectin, μg/mL	12.2 ± 7.3	7.4 ± 4.0 *	6.8 ± 3.5 *	<0.001
Adpn/Lep ratio	2.83 ± 2.16	0.74 ± 0.15 *	0.21 ± 0.11 *^,^**†**	<0.001

Data presented as mean ± SD. Adpn/Lep ratio, adiponectin/leptin ratio; M, male; F, female; BMI, body mass index; SBP, systolic blood pressure; DBP, diastolic blood pressure; HOMA, homeostatic model of assessment; QUICKI, quantitative insulin sensitivity check index; LDL, low-density lipoprotein; HDL, high-density lipoprotein; CRP, C-reactive protein; WBC, white blood cells; ALT, alanine aminotransferase; AST, aspartate aminotransferase; γ-GT, γ-glutamyltransferase. Differences between groups were analyzed by ANOVA followed by LSD tests. *p < 0.05 vs. ≥ 1.0. **†** *p* < 0.05 vs. ≥ 0.5 <1.0. Differences in gender distribution were analyzed by χ^2^ analysis. CRP concentrations were logarithmically transformed for statistical analysis.

**Table 2:** Contingency tables of the individuals classified according to the Adpn/Lep ratio and metabolic conditions.

	≥1.0	≥0.5 <1.0	<0.5	*p*
**Obesity (*n* = 292)**				<0.001
Lean	50 (60%)	29 (35%)	4 (5%)	
Obese	1 (1%)	21 (10%)	187 (89%)	
**Type 2 diabetes (*n* = 289)**				<0.001
Normoglycemia	40 (25%)	33 (21%)	84 (54%)	
Impaired fasting glucose	10 (10%)	13 (14%)	72 (76%)	
Type 2 diabetes	1 (3%)	4 (11%)	32 (86%)	
**Metabolic syndrome (*n* = 246)**				<0.001
Without MS	32 (27%)	31 (26%)	57 (47%)	
With MS	1 (1%)	11 (9%)	114 (90%)	

Data presented as number of subjects. Individuals were segregated according to the Adpn/Lep ratio proposed cut-offs: ≥1.0, ≥0.5 <1.0 and <0.5. Differences in distribution regarding the proposed cut-offs and each metabolic condition (obesity, type 2 diabetes or metabolic syndrome) were analyzed by χ^2^ analysis. Partial percentages within groups are shown. MS, metabolic syndrome.

**Table 3:** Analysis of the correlation between several anthropometric and cardiometabolic variables and the markers of adipose tissue dysfunction Adpn/Lep ratio and visceral adiposity index (VAI).

	Adpn/Lep Ratio		VAI	
Variable	*r*	*p* value	*r*	*p* value
Age	−0.05	0.430	−0.06	0.319
Sex	−0.04	0.542	−0.05	0.461
BMI	−0.49	0.000	0.30	0.000
Body adiposity	−0.56	0.000	0.27	0.000
Waist circumference	−0.51	0.000	0.35	0.000
SBP	−0.18	0.004	0.01	0.870
DBP	−0.35	0.000	0.13	0.037
Glucose	−0.19	0.003	0.23	0.000
Insulin	−0.22	0.000	0.31	0.000
HOMA	−0.24	0.000	0.36	0.000
QUICKI	0.36	0.000	−0.41	0.000
Triglycerides	−0.19	0.002	0.92	0.000
Total cholesterol	−0.09	0.157	0.28	0.000
LDL-cholesterol	−0.14	0.031	−0.02	0.791
HDL-cholesterol	0.30	0.000	−0.53	0.000
Uric acid	−0.27	0.000	0.37	0.000
CRP	−0.39	0.000	0.30	0.000
Fibrinogen	−0.39	0.000	0.22	0.020
Homocysteine	−0.01	0.885	−0.03	0.783
WBC	−0.27	0.003	0.18	0.053
ALT	−0.24	0.000	0.25	0.000
AST	−0.08	0.201	0.17	0.008
AST/ALT ratio	0.39	0.000	−0.23	0.000
γ-GT	−0.16	0.014	0.31	0.000
Creatinine	0.08	0.210	0.09	0.189
SAA	−0.18	0.034	0.10	0.245

Values are Pearson’s correlation coefficients and associated *p* values. CRP concentrations were logarithmically transformed for statistical analysis. Adpn/Lep ratio, Adiponectin/Leptin ratio; VAI, visceral adiposity index; BMI, body mass index; SBP, systolic blood pressure; DBP, diastolic blood pressure; HOMA, homeostatic model assessment; QUICKI, quantitative insulin sensitivity check index; LDL, low-density lipoprotein; HDL, high-density lipoprotein; CRP, C-reactive protein; WBC, white blood cells; ALT, alanine aminotransferase; AST, aspartate aminotransferase; γ-GT, γ-glutamyltransferase; SAA, serum amyloid A. For correlation with gender, male = 1 and female = 2 was used.

## Supplementary Materials

The following are available online at https://www.mdpi.com/2072-6643/11/2/454/s1, Figure S1: Adpn/Lep ratio according to body adiposity in the 83 lean subjects (low adiposity, *n* = 42; high SAA *n* = 41). Statistical differences were analyzed by two-tailed unpaired Student’s *t* test., Table S1: Analysis of the correlation between several anthropometric and cardiometabolic variables and the markers of adipose tissue dysfunction Adpn/Lep ratio and VAI after adjustment by BMI.
