# Supplementary material for: Adiponectin-leptin Ratio is a Functional Biomarker of Adipose Tissue Inflammation
Source: Nutrients. 2019 Feb 22;11(2):454. doi: 10.3390/nu11020454 (PMC6412349; doi:10.3390/nu11020454)
Supplement: Supplementary file 1 [file nutrients-11-00454-s001.pdf]

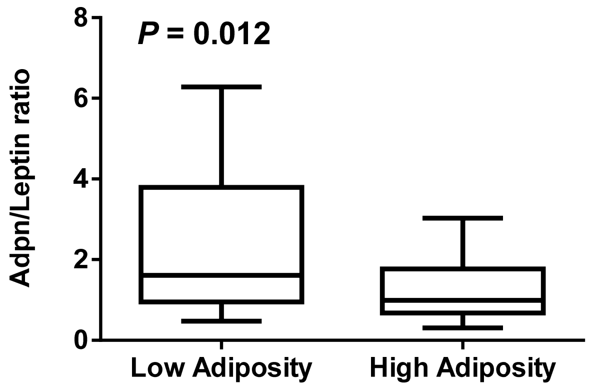

Figure S1. Adpn/Lep ratio according to body adiposity in the 83 lean subjects (low adiposity, n=42; high SAA n=41). Statistical differences were analyzed by two-tailed unpaired Student's t test.

Table S1. Analysis of the correlation between several anthropometric and cardiometabolic variables and the markers of adipose tissue dysfunction Adpn/Lep ratio and VAI after adjustment by BMI.

	**Adpn/Lep ratio**		**VAI**	
Variable	*r*	*P* value	*r*	*P* value
Age	-0.22	0.000	0.01	0.886
Sex	-0.04	0.484	-0.05	0.438
BMI	—	—	—	—
Body adiposity	-0.25	0.000	0.03	0.632
Waist circumference	-0.16	0.009	0.20	0.002
SBP	-0.15	0.012	-0.04	0.582
DBP	-0.35	0.001	0.03	0.680
Glucose	-0.06	0.276	0.17	0.009
Insulin	-0.03	0.610	0.21	0.000
HOMA	-0.04	0.506	0.27	0.000
QUICKI	0.13	0.034	-0.30	0.000
Triglycerides	-0.11	0.068	0.91	0.000
Total cholesterol	-0.15	0.011	0.30	0.000
LDL-cholesterol	-0.13	0.027	-0.05	0.431
HDL-cholesterol	0.06	0.323	-0.46	0.000
Uric acid	-0.12	0.040	0.28	0.000
CRP	-0.14	0.025	0.15	0.022
Fibrinogen	-0.15	0.067	0.05	0.606
Homocysteine	-0.05	0.530	-0.10	0.286
WBC	-0.19	0.033	0.13	0.142
ALT	-0.06	0.308	0.15	0.020
AST	-0.02	0.740	0.11	0.081
AST/ALT ratio	0.21	0.000	-0.13	0.050
γ-GT	-0.04	0.471	0.25	0.000
Creatinine	0.01	0.854	0.14	0.036
SAA	-0.15	0.056	0.05	0.554

Values are Pearson's correlation coefficients and associated *P* values. CRP concentrations were logarithmically transformed for statistical analysis. Adpn/Lep ratio, Adiponectin/Leptin ratio; VAI, visceral adiposity index; BMI, body mass index; SBP, systolic blood pressure; DBP, diastolic blood pressure; HOMA, homeostatic model assessment; QUICKI, quantitative insulin sensitivity check index; LDL, low-density lipoprotein; HDL, high-density lipoprotein; CRP, C-reactive protein; WBC, white blood cells; ALT, alanine aminotransferase; AST, aspartate aminotransferase; γ-GT, γ-glutamyltransferase; SAA, serum amyloid A. For correlation with gender, male=1 and female=2 was used.
